# XML-based e-assessment system for Office skills in open learning environments

**DOI:** 10.1186/s41039-015-0008-y

**Published:** 2015-07-20

**Authors:** Aicha Chorana, Abdallah Lakhdari, Hadda Cherroun, Slimane Oulad-Naoui

**Affiliations:** Laboratory of Computer Sciences and Mathematics, Faculty of Sciences, University of Laghouat, P.O. BOX 37G, Laghouat, 03000 Algeria

**Keywords:** E-assessment, Open learning, XML

## Abstract

Learning and teaching systems have seen fast transformations being increasingly applied in emerging formal and informal education contexts. Indeed, the shift to open learning environments is remarkable, where the number of students is extremely high. To allow a huge amount of learners gaining new knowledge and skills in an open education framework, the recourse to e-assessment systems able to cover this strong demand and respective challenges is inevitable. Facing Office skills as those most frequently needed in education and business settings, in this paper, we address the design of a novel assessment system for automated assessment of Office skills in an authentic context. This approach exploits the powerful potential of the Extensible Markup Language (XML) format and related technologies by transforming the model of both students’ documents and answers to an XML format and extracting from the teacher’s correct document the required skills as patterns. To assign a mark, we measure similarities between the patterns of the students’ and the teacher’s documents. We conducted an experimental study to validate our approach for Word processing skills assessment and developed a system that was evaluated in a real exam scenario. The results demonstrated the accuracy and suitability of this research direction.

## Background

In the information technology (IT) fast revolution age, there is a global demand for mastering IT skills. Mastering those skills refers to the ability of an individual to efficiently handle an operating system, a Word processor, a spreadsheet application, a Web browser, and an e-mail software, where the most popularized and frequently needed ones are Word processing and spreadsheet skills, especially in education and business settings. Both modern educational and professional organizations need to evaluate the IT competencies and skills of a large number of persons in many exams. Often, we can refer also to Open Learning systems, which constitute informal education scenarios and are increasingly in demand of many learners that are not able to enroll in formal educational institution arrangements. Consequently, many systems for certification and assessment of this kind of competencies and skills have emerged. Among the most well-known ones is the European Computer Driving License (ECDL; http://www.ecdl.org).

According to Lazarinis (2006), the origin of the ECDL certification goes back to 1994 through a European initiative which aims at the establishment of a certification for basic IT skills. At the beginning, that certification was called Computer Driving License (CDL); now it is named ECDL and is globally recognized as the certification which holds the larger market share compared to other certifications (Weiss and Sellin 2006). Lazarinis also reviewed other information and communication technology (ICT) certifications of private organizations and universities, such as the Microsoft Office User Specialist (MOS; http://www.certiport.com) and the Cambridge International IT Skills (http://www.cie.org.uk), and pointed out the importance of the ICT certifications as they make employees stay current with new technologies and help them in a job-relevant situation.

The demand for automated assessment and certification systems has grown due to the fact that it would be extremely complex and hard to assess the huge amount of candidates manually by humans. This demand is confirmed by both employability and institutional needs. This situation drives the design of automated assessment systems in order to decrease both cost and time of manual assessment processes. Furthermore, automated assessment and certification systems are expected to avoid subjectivity and errors of human assessment. Indeed, a panoply of systems and tools have been designed and implemented in order to automatically assess IT skills. The next section provides a detailed review of those developments.

As referred to by Mohammad and Gutl (2008), according to Martell (2005), the assessment process starts by identifying the learning goals and objectives. Consequently, e-assessment systems are strongly affected by this requirement. With regard to the consideration of learning objectives, Bloom’s taxonomy is frequently applied (Bloom 1956). It represents a classification of the learning objectives into six hierarchically ordered levels, namely knowledge, comprehension, application, analysis, synthesis, and evaluation. The learning objectives influence noticeably the type of the assessment system. Culwin (1998) distinguishes two kinds of assessment systems: (1) fixed response systems and (2) free response systems. In connection with fixed response systems or objective systems, a pre-prepared list of solution alternatives is available, where the user is forced to provide a fixed response by selecting an answer from that list, whereas in the free response systems or the nonobjective ones, the response of the user is formulated by unanticipated answers. In this latter type of systems, the higher Bloom’s learning objective levels are assessed, for example, skills like programming, essays writing, and meta-skills can be assessed rather than knowledge, which represents the main domain of fixed response systems. Thus, the most suitable assessment systems for IT skills refer to free response systems. Furthermore, the most efficient systems are those allowing an authentic assessment where the candidates are placed in a real-life or simulated scenario that requires them to apply accurate knowledge and skills (JISC 2007).

Against this background, we need to distinguish between the terms assessment and grading. In fact, assessment is broader than grading as it can provide feedback and some guiding principles for both teachers and students, which improves the learning process. In this paper, the terms automated assessment systems, automated grading systems, and automated marking systems are used interchangeably pointing out different studies in the literature, but when particular systems provide feedback, it is underlined.

Over the last few decades, new education forms have increased the student-to-lecturer ratios, as an example, the open education. Arnold (2012) regards the Massive Open Online Courses (MOOCs) as the best example to illustrate the issue of effectively assessing thousands of students enrolled in these courses each semester. Considering the assessment as an integral part of the learning process (Ehlers 2013), we can also transform this fact to learning systems and automated assessment. There is a major agreement that automated assessment systems are really motivating. Lahtonen and Isomöttönen (2012) argue that this motivation refers to the reduction of the teacher’s workload and the easiness of interaction between the teacher and students. They summarize that automated assessment can also make grading more consistent and objective. This fact has resulted in an increasing effort to develop automated grading systems in order to achieve these objectives. Although the research in the design of automated assessment systems has a long history in many educational domains (Lahtonen and Isomöttönen 2012), only a few attempts have been established to develop automated assessment systems for IT skills, and Office skills in particular (Kovacic and Green, 2012).

In this paper, we present a novel approach for assessing IT skills automatically. It is based on the Extensible Markup Language (XML) representation of both the teacher’s document and the students’ production by measuring their similarity to assign relevant marks. Then, our approach is illustrated by a grading system for the case of assessing Word processing skills. The automated assessment system, we describe in this paper, is a part of a framework to assess Office skills that we currently design and develop. In the “Related work” section, we present the related work both for automated assessment in general and for IT skills in particular. “The novel e-assessment approach” section outlines our approach for automated assessment of IT skills and how it can be generalized to assess other skills and in other domains of knowledge. The prototype implementation details and experiments are summarized and reported in “Evaluation framework” section. Finally, concluding remarks and suggestions for future work are presented in the “Conclusion and future directions” section.

## Related work

The history of e-assessment goes back to the 1960s (Mohammad and Gutl 2008). This history, elaborated by many authors as an example of the recent ones by Jordan (2013) as well as Lahtonen and Isomöttönen (2012), shows that many assessment systems have been developed for many knowledge domains, such as mathematics, programming languages, and free text. However, a few attempts have led to sufficient grading systems for automated assessment of IT skills, especially for Office skills (Kovacic and Green 2012) despite the significant need identified. In general, there are two strategies for automated assessment of Office skills (Zhu and Shen 2013; Tang and Chen 2008). The first strategy applies the trace analysis technique. It records the operating steps of the users, for example by using the macro recording function of Microsoft Office. Another example addresses the construction of a simulation system, which is considered as a large project and has as drawbacks the difficulty of updating the software, the consideration of the environmental constraints, as well as the poor adaptability. Dowsing (2000) proposed a system that performs a comparison between the stream of actions done by the candidate and the correct stream of actions extracted from the model event stream of the teacher. The second strategy, most natural, consists of analyzing directly the student’s produced document. This strategy has been frequently implemented in contrast to the first strategy (Zhu and Shen 2013; Tang and Chen 2008). Evidently, this direction of development, maintenance, and extension is less problematic since it is not constrained by the operating environment and, generally, it reflects the students’ proficiency in Office applications more realistically.

**Fig. 1:**
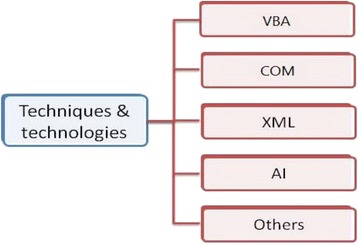
Techniques and technologies applied in Office skills assessment systems

Following this line, we concentrate in this study only on related work that follows this latter strategy. In general, there are many practical implementations to mark a student’s outcomes by analyzing the student’s produced document.As illustrated in Fig. 1, we have classified these developed systems according to the techniques and technologies they apply. These techniques and technologies are the following: (1) the Visual Basic for Application (VBA), (2) the Component Object Model (COM), (3) the XML technology, and (4) Artificial Intelligence (AI) techniques and other techniques such as using the “Combine and Compare” feature of Microsoft Office Word.

In the first category, assessment systems use the VBA technology, which is a version of the Visual Basic language included in Microsoft Office. VBA enables the user to design routines that will run in the background in order to respond to events such as opening a form or clicking a command button (Herbadji 2012). In addition, the VBA technology allows an easiness of communication with Office programs. According to the literature review, most of the developed assessment systems for Office applications rely on the VBA technology (Koike et al. 2005; Tuparova and Tuparov 2010; De-cai 2010; Wang and Jing-zhu 2009; Ren et al. 2010). For example, Koike et al. (2005) developed marking systems for Microsoft Office Word and Microsoft Office Excel. The systems mark student files according to a grading criteria set given by the instructors. The system for Word files checks page settings, paragraphs, indents, figures, tables, fonts, colors, texts, and so on. The system can be applied to check whether the students correctly understand how to use each feature of Microsoft Office Word. Similarly, for Excel files, the system can be applied to check whether students understand how to use each feature of Microsoft Office Excel. Both systems highlight errors and students get feedback on their work through messages. Systems in this category are not flexible to modify or add new questions. They require VBA programming abilities as confirmed by Tuparova and Tuparov (2010) who advanced such systems by proposing a real-live performance-based assessment tool using VBA and allowing for an effective interaction with the teacher.

Concerning the second category in Fig. 1, assessment systems adopt the COM technology, which is a binary-interface standard for software components introduced by Microsoft (https://www.microsoft.com/com). It is used to enable software components to communicate with each other and serves as a basis for many systems (Zhu and Shen 2013; Hunt et al. 2002; Tang and Chen 2008). For example, Zhu and Shen (2013) present a framework implementation of automated assessment for Office applications. The system stores the required IT skills in a database table and invokes a series of methods through the COM interface provided. These methods simultaneously extract the attributes of the students’ documents, match them with information from the database, and grade according to the scoring criteria. Zhu and Shen (2013) argue that using the COM technology results in the problem of a complete analysis of the Office object library. Viewed this way, this technology shows limitations and does not supply assessment for all skills.

Recently, Roshan et al. (2011) and Lahtonen and Isomöttönen (2012) present work with respect to a different format than the Office one. Lahtonen and Isomöttönen (2012) developed the Parsi Tool by exploiting the XML technology. Their main goal is to automatically assess the stylistic and technical correctness of Office documents as well as some basic IT skills, such as e-mail netiquette and e-mail list usage. The Parsi Tool has as an input the student’s document in a set of specific format (e.g., docx, pptx, xls). It also has an XML configuration file which represents the requirements of the assignments. This file contains the required style information for Word processing and presentations and further checkable items, such as Bold and Page numbering skills. As output, the Parsi Tool returns a grade from 1 to 5. The teacher may intervene when the tool cannot check properly, by updating and testing the checking functions.

According to Fig. 1, some other systems apply AI techniques (Dowsing 1996; Dowsing 1998; Long et al. 2003). Dowsing and Long set a milestone for latter works on the assessment of Word processing skills being pioneers in this domain since 1996. They exploited AI techniques to assess the produced Word documents. The software developed to assess Word processing skills is a part of a project to assess other IT skills by a computer. It is based on the assessment of Rich Text Format (RTF) output from any standard Word processor. A comparison is performed between the examinees’ outputs and the teacher’s correct solution, followed by a categorization and error report by type. Also, Long et al. (2003) describe a set of rule-based methods and knowledge-based automated assessment systems for IT skills.

Related to the last category “Others” in Fig. 1, Hill (2011) developed several tools for automated grading in terms of Microsoft Office software, particularly an automated assessment system for Microsoft Office Excel (Microsoft Excel Automated Grader—MEAGER) and an automated assessment system for Microsoft Office Access (Microsoft Access Database Automated Grading System—MADBAGS) (Hill 2004). Recently, his focus is on Microsoft Office Word and Microsoft Office PowerPoint programs. These grading systems exploit the Microsoft Word “Compare and Combine” function. This latter feature of Microsoft Office Word allows systems to merge documents to identify differences between them. Thus, the document of the correct version of the assignment given by the instructor is merged with the document produced by the student, and the differences are then recorded in a Microsoft Office Access table. The Word Grader counts obtained errors and embeds a grade report in the marked-up document. Even though Hill’s developments are powerful, they are limited by the use of Microsoft Office programs only, and as the author stated, the ability to assess new skills (e.g., manipulating text boxes) that are not compared by the Microsoft Word “Compare and Combine” function will be more difficult.

Although the reviewed systems provide an authentic assessment, most of them lack flexibility in allowing teachers to customize exams by modifying questions or adding new ones, and defining their own grading criteria. After the thorough investigation of the state of the art in Office assessment systems, we argue that most of the examined systems implement the VBA technology, obviously, due to its inherent capability to build and communicate with Microsoft Office applications. However, it provides limited options for adapting the assessment process like modifying questions, which requires VBA programming skills by the teacher. A further drawback of the systems observed is the missing option for a human intervention in ambiguous and difficult assessment cases. Against this background, we focus on the XML format and its related technologies in the following.

## Methods

### The novel e-assessment approach

The main idea of our approach is to provide and popularize a simplistic Fully Automated Assessment Approach (F3A) for Office skills through a standard representation using the XML format (see below). Accordingly, the system can easily operate on both the student’s produced document and the teacher’s correct document without depending on any environment constraints.

### The advantage of an XML implementation

The Extensible Markup Language (XML) allows the representation, storage, and exchange of data because of its inherent integration of structural and semantic aspects of the document content. In addition, XML is very flexible due to its ability to encode any type of information, for example the Mathematical Markup Language (MathML) represents mathematics formulas, the Scalable Vector Graphics formats (SVG) two-dimensional graphics with support for interactivity and animation, and the Office Open XML corresponds to spreadsheets, charts, presentations, and Word processing documents (Carton 2010). Concerning this latter format, Microsoft started using XML for the Office Suite, in 2000, and adopted this format definitively in 2003 after many standardization efforts. It is possible to save Microsoft Office (for example Word, Excel, and PowerPoint) documents in XML format. Our particular driving force to use the XML format for the envisaged automated assessment is backed up by many aspects, such as:

1. Any electronic document can be represented using the XML format.
2. Many applications allow exporting their outcomes in XML format.
3. A significant amount of XML related tools (e.g., XQuery, XPath, DOM) exist.
4. It represents a textual, not binary format. Thus, it easily supports the extraction of useful information from documents.
5. XML is an open standard with interoperability features.
6. The associated mechanisms XML Schema Definition (XSD) and Document Type Definition (DTD)^1^ specify how to formally describe the elements of an XML document. They can be used by programmers to verify each piece of an item content in a document.
7. The suitability for integration in open learning environments.

Derived from these explanations, the next subsection provides insights on our XML-based approach to an automated assessment system.

### The F3A model

**Fig. 2:**
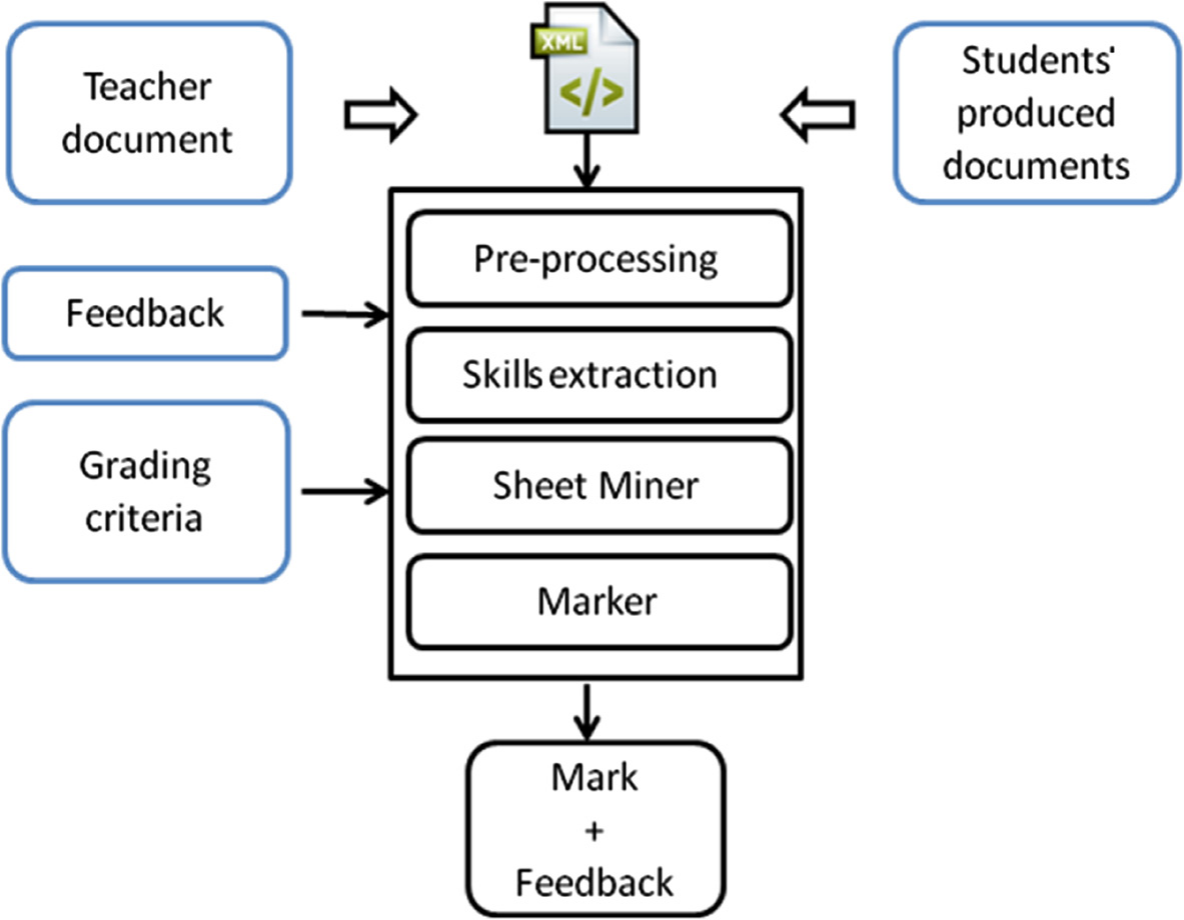
Global view of the F3A model

In this subsection, we present in detail the automated assessment approach. Figure 2 illustrates the F3A model where the following entries serve as an input: students’ produced documents, the grading criteria, the feedback, and the teacher’s correct document. This latter document is used to extract the required skills. The individual steps of the approach address the following processes:

1. Pre-processing
  As mentioned above, we strongly refer to the potential of XML and related technologies. At this step, all of the entries have to be transformed to the XML format. According to the application needs, the transformed entries are parsed to get their tree structure. This step can also perform some cleaning tasks to get more readable XML trees consistent with the required skills.
2. Skills extraction
  While each event or action of any IT function can be stored in the XML representation (for example, producing Word or spreadsheet document, preparing PowerPoint presentation, system configuration, etc.), at this step, the required skills are extracted from the XML tree representation of the teacher’s correct document. Each skill is described by a path expression.
3. Sheet Miner
  A similarity measure is applied between the expressions of all solicited skills extracted from the teacher’s correct document and those extracted from the XML tree representation of the student’s produced document (students’ answers). One can consider this step as a tree pattern matching task.
4. Marker
  According to the results of the similarity measurement provided by the previous step, a grade will be accredited for each skill, following the predefined grading criteria. In addition, a feedback is provided for a no performed skill.

Following the description of the F3A processes, the next subsection deals with the F3A applicability and its possible generalization to Office skills and other knowledge domains.

### Discussion

The explanations provided in the previous subsections confirm the valuability of the XML-based approach toward its built-in generalization potential for the assessment of any IT skills with regard to Office programs, such as spreadsheets and PowerPoint. Since the starting point of this research is to compare two XML documents, and in addition, all Office programs can be saved in the XML format, it is evidently possible to customize automated assessment systems to fit to the assessment of further Office skills.

Moreover, this methodology can be also applied to any other IT skills, such as system configuration for example. Moreover, it can be applied to any other domain, when it is possible to capture or transform answers in an XML format. Indeed, the DTD mechanism allows the teacher to define the elements/attributes associated to the targeted skills. Thus, the final user is free to customize the DTD according to the assessment requirements.For instance, if the assessment targets a computer science exam, such as Linux Operating System and its external commands, one can define a DTD by associating “Linux commands” to “Elements”, and “Options” to “Attributes”*.*

Facing the generalization opportunity for the F3A approach that targets at the most used business and education application, the Office Suite, the next section presents the performance evaluation of the described F3A approach and offers discussion of the results achieved.

### Evaluation framework

In order to measure the performance of the XML-based approach, we apply it in a real-life exam, moreover, in such a way that the scenario can be closely mapped onto open learning requirements to fulfill informal learning needs. Due to the wide usage of Microsoft Word applications, we especially target at the assessment of Word processing skills. There are two ways to assess those skills. The first way represents a face-to-face method, where the instructor in person checks the learner’s skills, whereas the second way involves the analysis of the student’s produced document. And since open learning environments are driven by online solutions, the produced documents for the assessment are submitted online. Toward the online adoption, the implemented algorithms of the system (teacher Graphical User Interface (GUI), extraction, and grading modules) are realized with Java for a fast prototyping and easy interoperability with XML tools. In the following, the explanations provide details on the system developed, the sample used to evaluate its performances, as well as the results achieved.

### Description of the automated assessment system

**Fig. 3:**
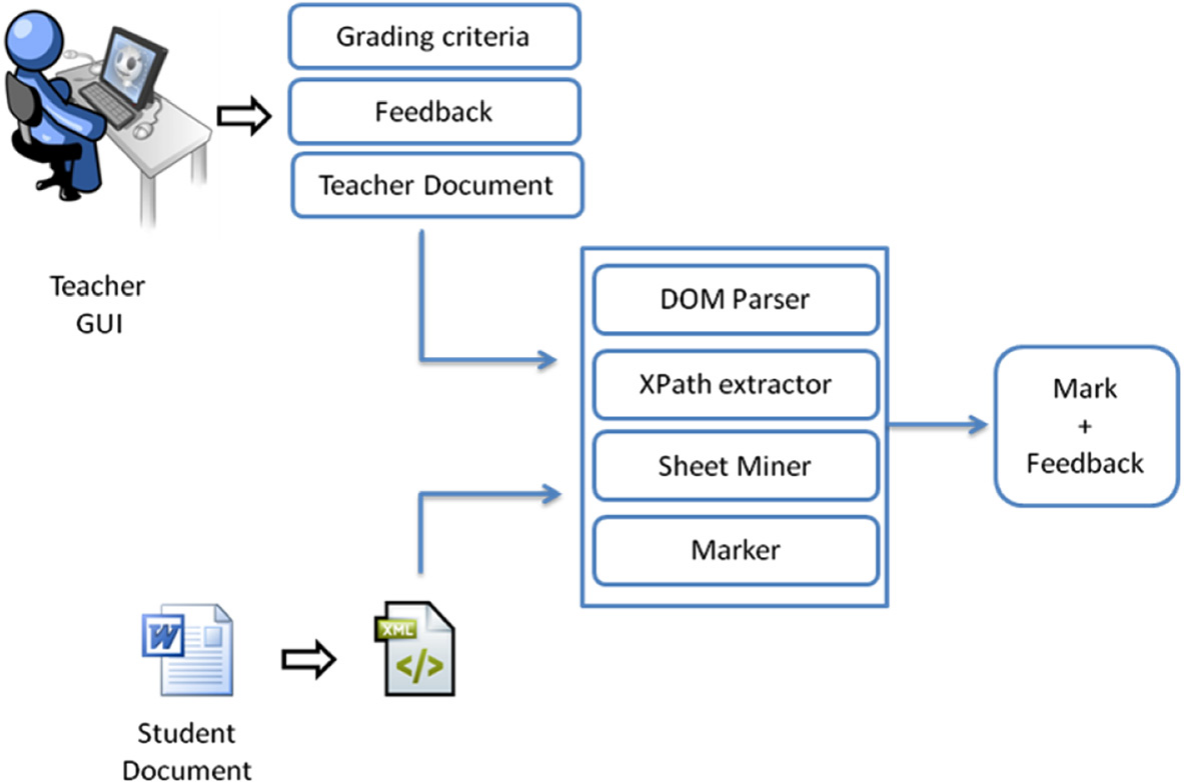
System structure

**Fig. 4:**
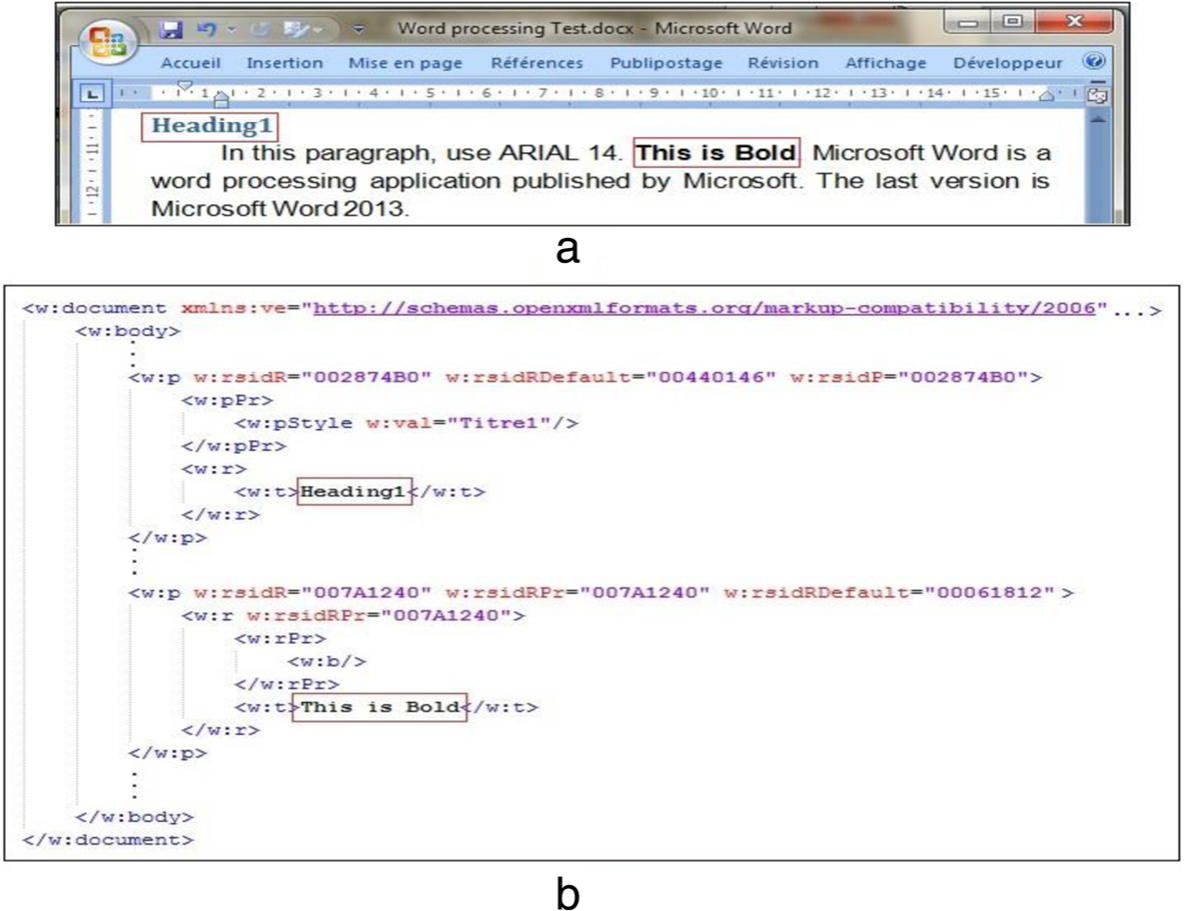
(**a**) A part of a Word document and (**b**) its XML representation of some required skills

In this subsection, we provide detailed explanations of the developed system to assess Word processing skills. Figure 3 illustrates the structure and functionalities of the automated assessment system. All entries are represented by the use of the Office Open XML format (TC45 and Ngo 2008). Figure 4a illustrates a part of a student’s produced document where the red rectangles denote two required skills, namely Bold and Heading style skills. Figure 4b demonstrates the respective XML format.

The implementation details of each module are described in the following by using an example to illustrate the outcomes of each step.

- Teacher GUI

The system is supported by a user-friendly interface in order to simplify the teacher’s tasks, such as elaborating exams, adding the grading criteria, defining feedbacks, etc. This interface does not require any additional workload from the teacher with respect to programming intervention or defining configuration files like the XML configuration file in the Parsi Tool (Lahtonen and Isomöttönen 2012). The teacher provides the students’ produced documents, the correct document, the grading criteria, and the feedback. As an example of a possible feedback, the case where heading style skill is not performed by a student, the following feedback was generated:

- In Microsoft Word 2007/2010, select the text to be used as a title or heading.
- On the Home tab, click the “Styles” button.
- Click the appropriate Heading style (for example, Heading 1, Heading 2, Title, Subtitle, etc.) to apply the style to the selected text.

In the current version of the automated assessment system, the teacher provides feedback only when the skills are totally missed.

- Document Object Model (DOM) parser

Many Application Programming Interfaces (APIs) exist to parse XML documents. The most known are Simple API for XML (SAX; http://www.saxproject.org/) and DOM (http://www.w3.org/DOM/). SAX is an event-based API, whereas DOM is a tree-based API, which creates a hierarchical data structure, a tree structure corresponding to the XML document. Thus, we adopt the XPath language which works with tree structures to extract information from the XML document, and the DOM API. Standardized by W3C, DOM is a cross-platform and language-independent convention for representing and interacting with objects in XML documents. The objects in the DOM tree are addressed and manipulated by applying methods on the objects. The DOM parser provides a tree structure for the XML format of the student’s and teacher’s Word documents to serve the next steps of the assessment process.

**Fig. 5:**
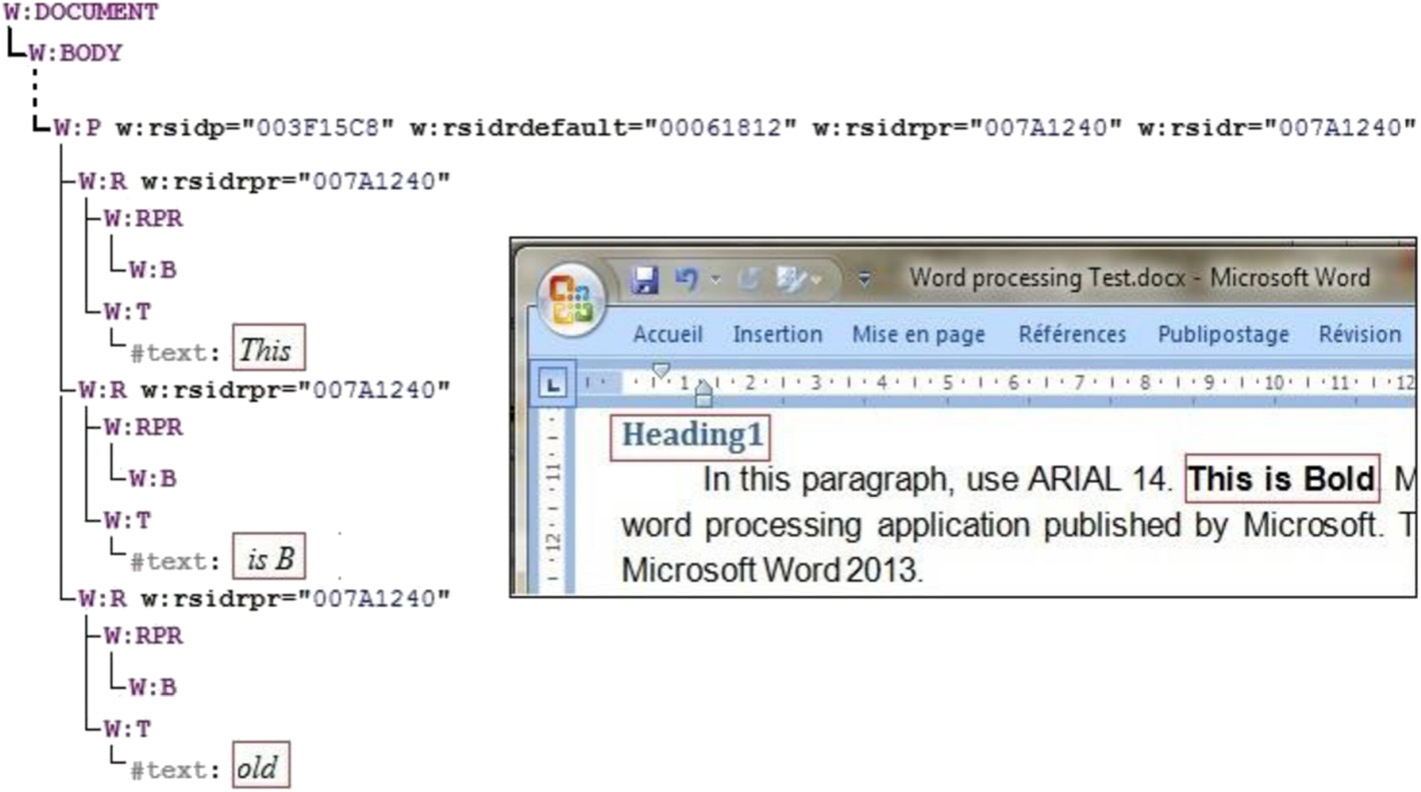
Example of a no pre-processed DOM tree

**Fig. 6:**
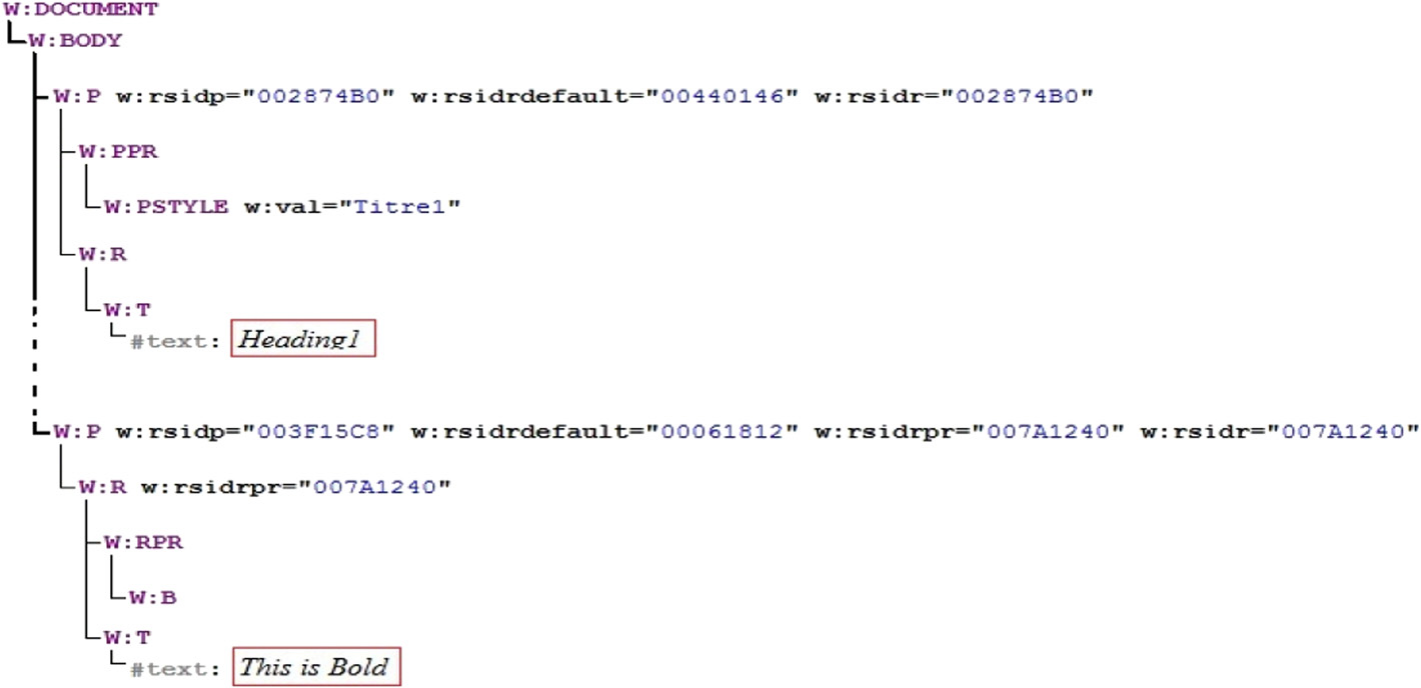
DOM tree of the XML presentation of Bold and Heading skills

With reference to our illustrative example, Fig. 5 shows a DOM tree of the XML excerpt in Fig. 4b that resulted from the DOM parsing. In order to obtain a cleaner tree, the resulted DOM tree is pre-processed by accomplishing the following two steps:

1. Cleaning empty elements: An empty element could be an empty paragraph, where all tags with their attributes but no text can be found. Obviously, it is needless to treat this empty element, so it is removed.

2. Combining similar elements: We find many contiguous similar elements as shown in Fig. 6. These elements differ in texts only, whereas all their tags and attributes are equal. This problem may happen when students performed many intermediate “save” actions when writing the text, for example the text “This is Bold” is cut out in three slices due to several “save” actions.

- XPath extractor

This is the main module of the automated grader. Its name results from using the XPath expression language. This language allows processing values that comply with a data model, which provides a tree representation of the XML documents (Berglund et al. 2010). The result of an XPath expression is a selection of nodes from the input documents, or an atomic value, or more generally, any sequence allowed by the data model. It offers the ability to navigate around the tree and select nodes by a variety of criteria. We exploit the features of this technology, which allows the interrogation of an XML document. The aim is to extract the set of required skills (SRS) from the DOM tree of the teacher’s correct document. The elements of SRS can be regarded as patterns to be found in the student’s produced document. These patterns are described by their XPath expressions as presented in the following according to our example:

- Heading’s XPath expression:
  - //w:p[w:pPr/w:pStyle[@w:val='Titre1'] and./w:r/w:t[contains'Heading1')]]
- Bold’s XPath expression:
  - //w:(text(),p[w:r[w:rPr/w:b]/w:t[contains(text(),'This is Bold')]]

- Sheet Miner

This step serves the verification of the student’s ability to apply required Word processing skills. The Sheet Miner tries to find similarities between SRS elements (patterns) and existing patterns in the DOM tree of the student’s XML document. Accordingly, the main function of this module is to perform the pattern matching process, which can be done simply by evaluating the XPath expression of the required pattern included in SRS and using the DOM tree of the student’s produced document. Due to the specificity of the Word processing application and the XML format, two kinds of similarities can be calculated, which refer to two kinds of an assessment system:

- Structure similarity only: coarse-grained system
- Structure and content similarity: fine-grained system

In terms of this classification, the coarse-grained system considers similarity of structures only, whereby the skill is performed without strong requirements. This is more practicable in our case, which requires the student to apply some Word processing skills on specific text fragments, where spelling mistakes that can be committed by the students will cause the problem of different contents (strings) and correspond to unmatched patterns. Consequently, this fact leads to worse results in pattern matching, which influences the accredited marks. Concerning the fine-grained system, the similarity requires 100 % string matching. In our assessment framework, to deal with the issue of unmatched strings, we apply the bag of words technique, which means that we do not need the matching of the whole text fragment. Thus, the system developed can be referred to as a mixed-grained system, since it uses the structure similarity and partially the content similarity to improve the automated assessment results.

- Marker

This final step focuses on supplying the students’ marks and the feedback. A mark is assigned to each existing Word processing skill according to its similarity with the required one. As mentioned above, a feedback is offered when students are not performing skills. This feature corresponds to a formative assessment, which serves and improves the students’ learning processes.

The detailed explanation of the system step by step goes hand-in-hand with the targeted IT skills presented in the next subsection.

### Targeted skills

This subsection provides an overview of the most widely used Office skills, namely the Word processing skills, as defined in the ECDL Syllabus (ECDL 2007):

- Document creation
  - Open, close a Word processing application. Open, close documents.
  - Create a new document based on default template.
  - Save a document to a location on a drive.
  - Save a document under another name.
- Document formatting
  - Insert headers and footers.
  - Add fields in headers and footers.
  - Add automatically page numbering.
- Formatting characters
  - Enter Text, Select, Edit.
  - Display, hide nonprinting formatting marks like spaces.
  - Insert symbols or special characters like © and ™.
- Formatting paragraphs
  - Change text formatting: font sizes, font types.
  - Apply text formatting: bold, italic, underline.
  - Apply different colors to text.
  - Add bullets and numbers in a list.

Evaluating these skills provides information about the student level of Word processing abilities. The evaluation procedure applied and the results are discussed in the following subsections.

### Description of the sample

The evaluation of the automated assessment system developed was conducted in a real-life exam scenario with first-year graduate students approaching a Mathematic and Computer Science License (L1). A sample of 100 students, 56 % females and 44 % males, participated in an authentic exam with a duration of 1 h and graded on 10 points. In this exam, the students received printed instructions with the required skills and used the Microsoft Office Word 2007 program to create a Word document following the instructions. The produced documents were submitted to the instructor to be assessed.

## Results and discussion

The evaluation procedure involved three tests: (1) comparison between human assessment performed by three different teachers, (2) comparison between final marks obtained by human and automated assessment system, and (3) comparison between human and automated assessment system toward the identification of marks’ gaps by particular skill.

**Fig. 7:**
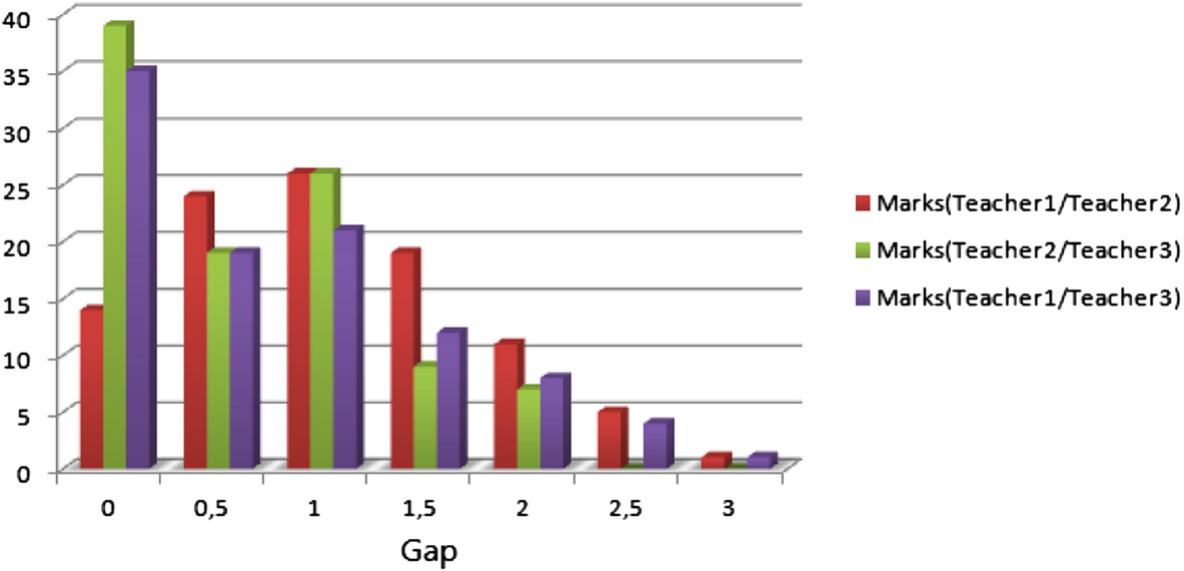
Distribution of students’ marks according to the gap in human assessment introduced by Teacher1, Teacher2, and Teacher3

The first test is performed in order to divulge some of the human assessment drawbacks, such as subjectivity and inaccuracy. For example, we compare marks assigned by three different teachers, Teacher1, Teacher2, and Teacher3. Figure 7 illustrates the differences between human assessment introduced by the three teachers. Each colored stick shows the ratio of marks given by two teachers having the gaps 0, 0.5, 1, etc. The differences between teachers’ assessments are obvious, namely only 29 % of the students’ marks are equal for all teachers.

**Fig. 8:**
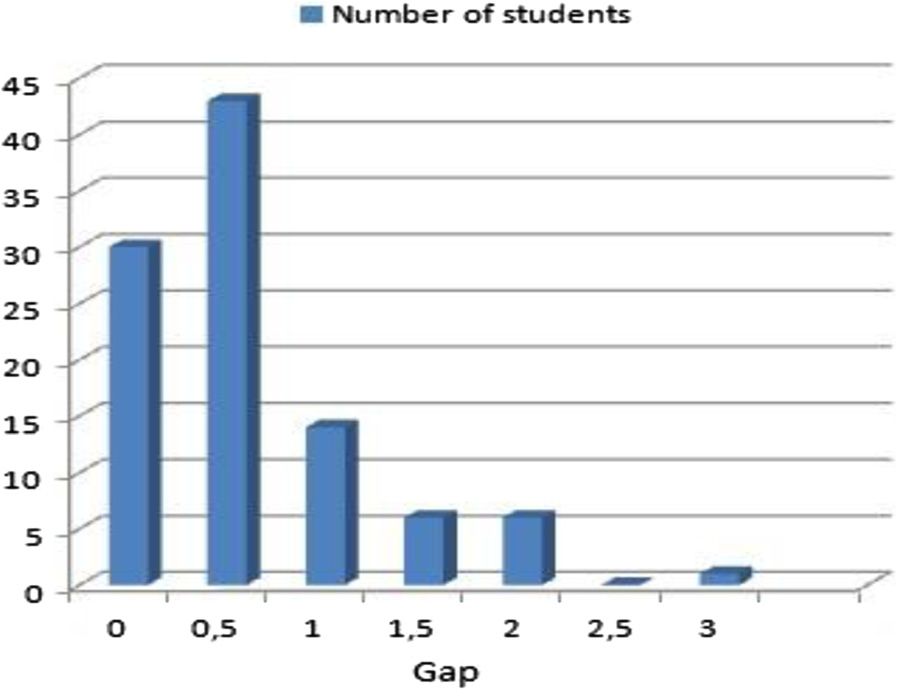
Distribution of students’ marks according to the gap between automated and human assessment

Figure 8 shows the distribution of students’ marks according to the gap value between the human assessment (average) and those obtained by the system developed. These results demonstrate that the automated assessment is very close to the human assessment: Less than 1 % has deviated by more than 2 points, compared with the human grading. Furthermore, 87 % of the students hold a mark with a gap between 0 and 1 points which is an acceptable result when assessing practical skills. Some deviations are caused by a degree of subjectivity and inattention of the teacher who assesses. This fact supports the notion of accuracy of the automated assessment accomplished with the developed system.

By analyzing the student’s document where the gap is 3 points (see Fig. 8), we found that the issue is caused by spelling mistakes when the required skill concerns “Editing a text *t* in a specific style” and a match is missing between the “input string” of a student with the required string or text *t*, which affects the result of the evaluated XPath expression.

**Fig. 9:**
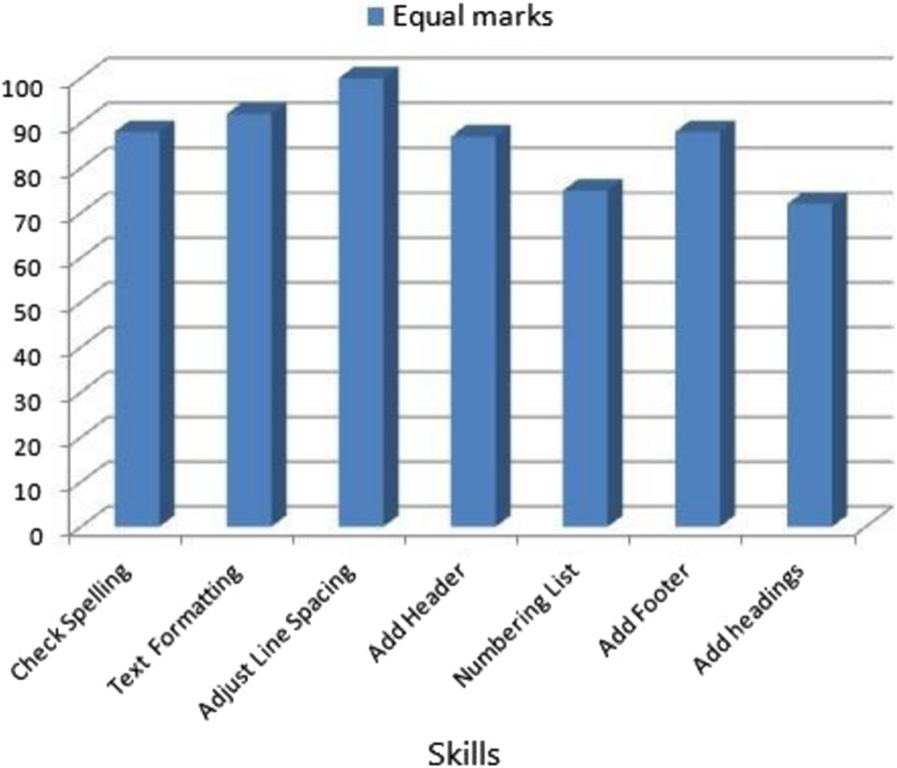
Similar automated and human assessment ratio by skill

Figure 9 illustrates the ratio of similar marks between the automated and human assessments by skill for a sample of seven skills. The aim of this measure is to spot cases when the automated assessment behaves differently from the human assessment. This figure confirms that for most of the skills, the ratio has exceeded 83 %. However, the skills “Numbering list” and “Add headings” have a ratio around 70 %. By investigating the origins of these values, we have depicted that the issue is caused by the inaccuracy of human assessment when too many details have to be assessed.

## Conclusion and future directions

In this paper, we have presented an approach to assess Office skills automatically evaluating the research in an authentic exam scenario. The automated assessment system adopts the XML format and its related technologies. It encompasses as an input the students’ produced documents, the teacher’s correct document, feedback, and the grading criteria. All entries are represented in an XML format in order to simplify their manipulation and the interoperability of the solutions. A set of tasks are applied in order to mark the student’s document, namely pre-processing, skills extraction, sheet mining, and marking.

In order to validate the assessment approach, we developed an automated assessment system for Word processing skills using the Microsoft Word program. The evaluation results show that the grader system marks are very close to the ones following from human assessment with reference to the benchmarks applied. Based on simplicity and transparency, this approach can be generalized to other Office and especially IT skills, such as system configuration. Moreover, while it is possible to capture answers in an XML format, our approach can simply be transferred to other domains of knowledge by defining the appropriate DTD to a particular exam, achieving in this manner a high level of generalization. In following this line, the exploitation of XML and related technologies in the development of novel e-assessment systems is the main result of this research offering an effective basis to serve a variety of emerging open education forms, such as Massive Open Online Courses (MOOCs) as well as support open learning solutions in terms of e-learning and learning as well as learner management systems.

The promising results, especially concerning the similarity measurement step, deliver ideas on how to extend our approach. In this emerging research, the required skills presented in the student’s production document have been taken into account and graded. However, no penalty is applied when the student gives more than what the teacher has asked. This consideration will direct our future work. Further, the recent version of the system effectively provides feedback to students. This helps in enhancing student learning. Moreover, giving reports and feedback to teachers about their students’ weakness in some skills can improve the content of their program and their way of teaching.

A further future work will address the extension of the automated assessment to address additional Office and IT skills, and improve the system performance by adding the possibility of automatically providing feedback based on former feedbacks to reduce the teacher’s workload and also investigate the application of data mining techniques like clustering similar XML documents to divide students’ documents into groups having similar or close answers.

## Footnotes

1 Both of them are recommendations of the World Wide Web Consortium (W3C).
